# Giant Vesicovaginal Calculi Secondary to a Forgotten Vaginal Tampon Complicated With Vesicovaginal Fistula: A Rare Case Report

**DOI:** 10.7759/cureus.106040

**Published:** 2026-03-28

**Authors:** Kramo N Felicite

**Affiliations:** 1 Urology, Université Félix Houphouët-Boigny, Abidjan, CIV

**Keywords:** bladder, calculi, cystolitotomy, fistula, vagina

## Abstract

Vesicovaginal fistulas in developing countries are mostly of obstetric origin. The etiology involving calcified vaginal foreign bodies is rare. We report an exceptional case of a giant vesicovaginal stone in a 67-year-old woman, secondary to a forgotten and calcified vaginal tampon, complicated by a vesicovaginal fistula. Treatment consisted of stone removal and fistula repair during the same surgical procedure as part of a health campaign for the treatment of urogenital fistulas. The postoperative course was uneventful; the patient was dry upon catheter removal.

This observation shows the importance of clinical diagnosis in situations of insufficient technical facilities and the feasibility of treatment in a single surgical procedure.

## Introduction

Urogenital fistulas have multiple causes. Among them, vesicovaginal fistulas are the most common type of acquired fistula. In developing countries, the causes are mostly obstetric [1]. Other etiologies, including radiation-related, tumor-related, infectious, traumatic, or iatrogenic, are also described in the literature. Fistulas secondary to the presence of prolonged calcified vaginal foreign bodies are rare [2]. The nature of vaginal foreign bodies varies: they can include pencils, batteries, staples, coins, toys, intrauterine devices, gauze pads, and even pieces of nylon, on which stones can develop and, more rarely, a vesicovaginal fistula [2,3]. We report the case of a 67-year-old patient who, more than 20 years after a vaginal delivery, presented with a vesicovaginal fistula due to an unrecognized vaginal tampon that had calcified.

## Case presentation

The patient was a 67-year-old woman admitted as part of a vesicovaginal fistula health campaign. She reported persistent urinary leakage from her vagina for over 10 years. Her medical history revealed a vaginal delivery more than 20 years prior, during which a vaginal tampon made of gauze pads had been inserted to control bleeding and then forgotten.

**Figure 1:**
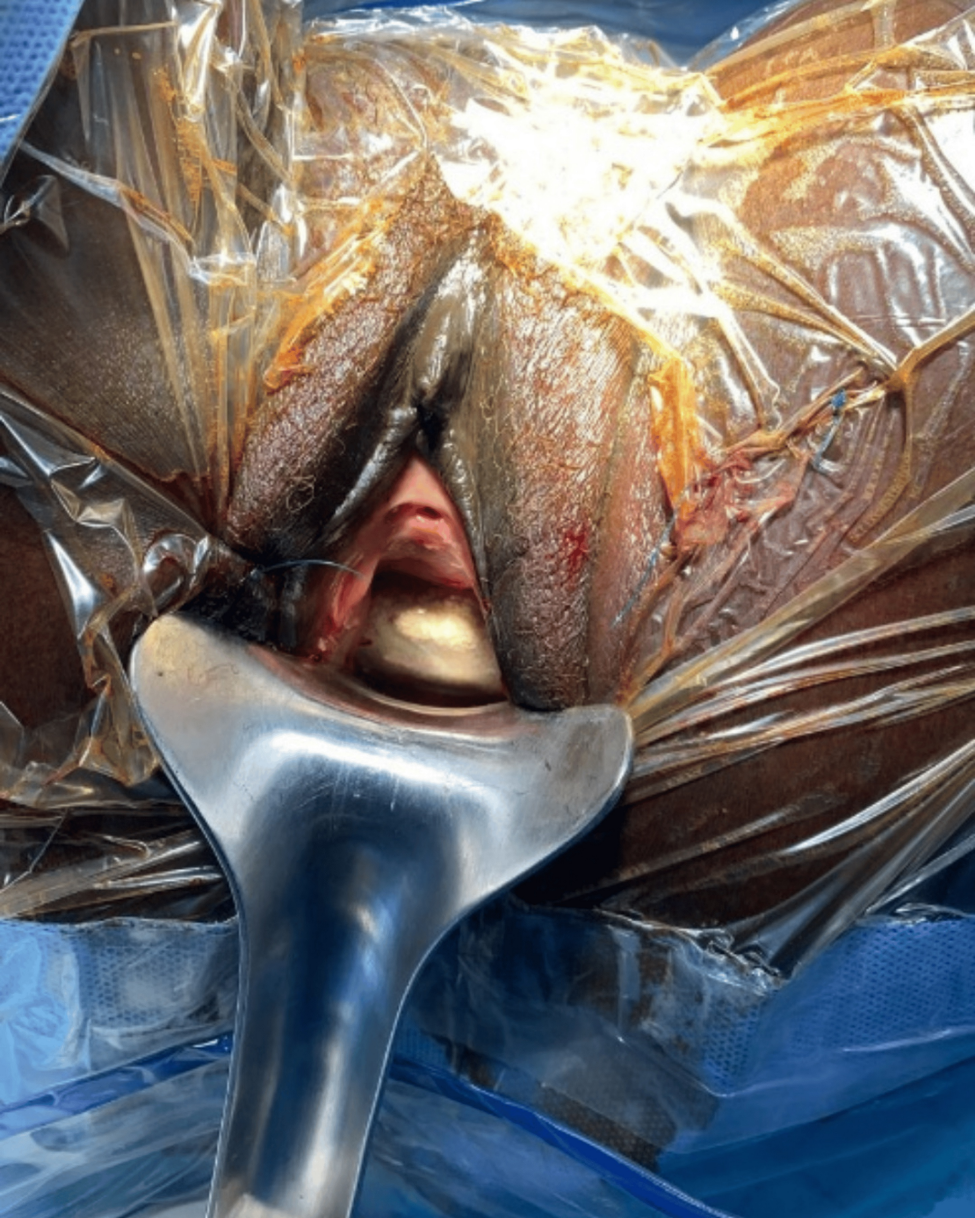
Preoperative image of a large vesicovaginal stone with difficulty in placing the weighted valve.

Clinical examination revealed a hard mass occupying the anterior vaginal wall. Exploration uncovered a large vesicovaginal stone associated with a retrotrigonal vesicovaginal fistula (Figure 1). No imaging was performed due to the clear clinical diagnosis and the context of a surgical campaign in a resource-limited setting.

**Figure 2:**
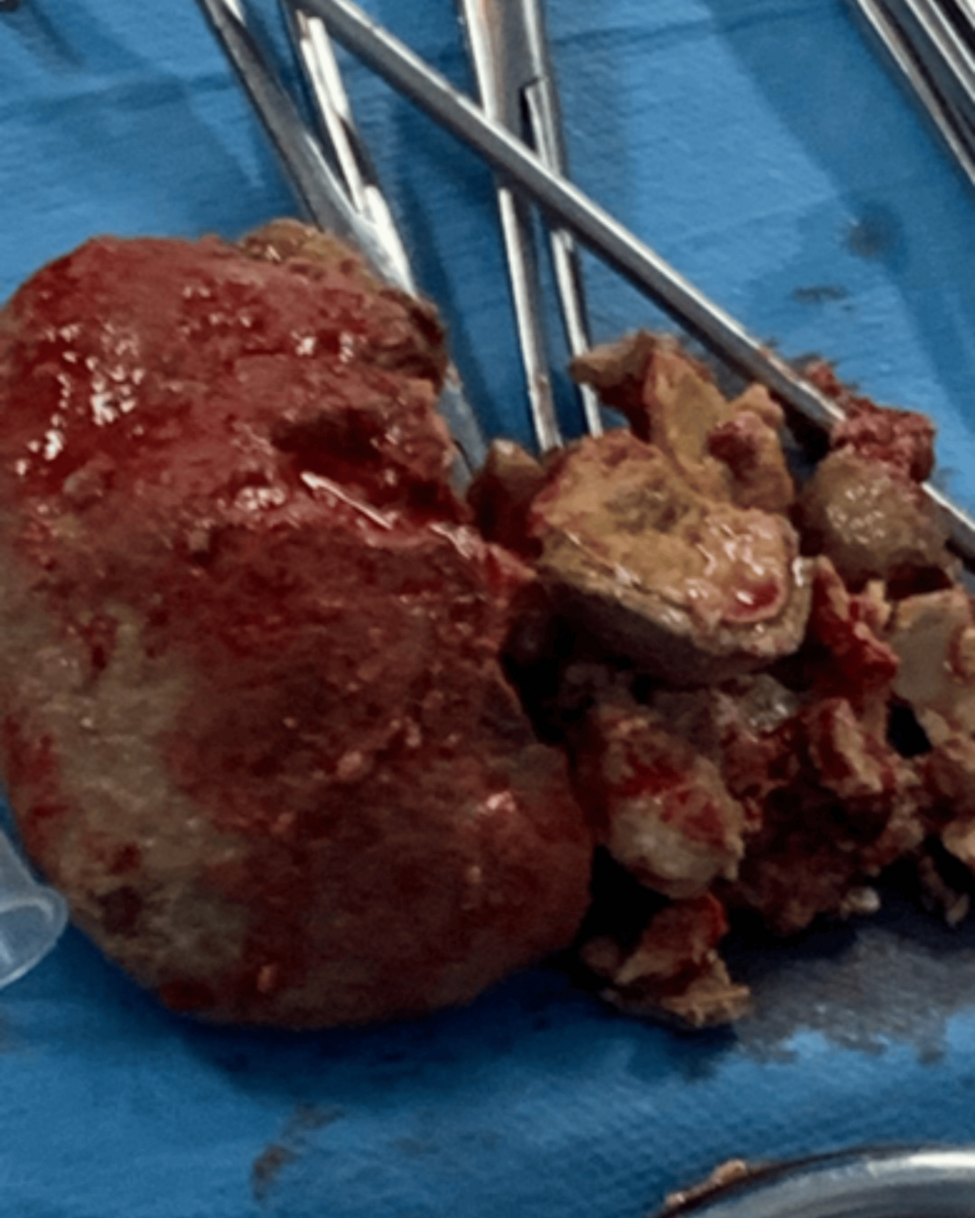
Giant vesicovaginal calculi after extraction.

**Figure 3:**
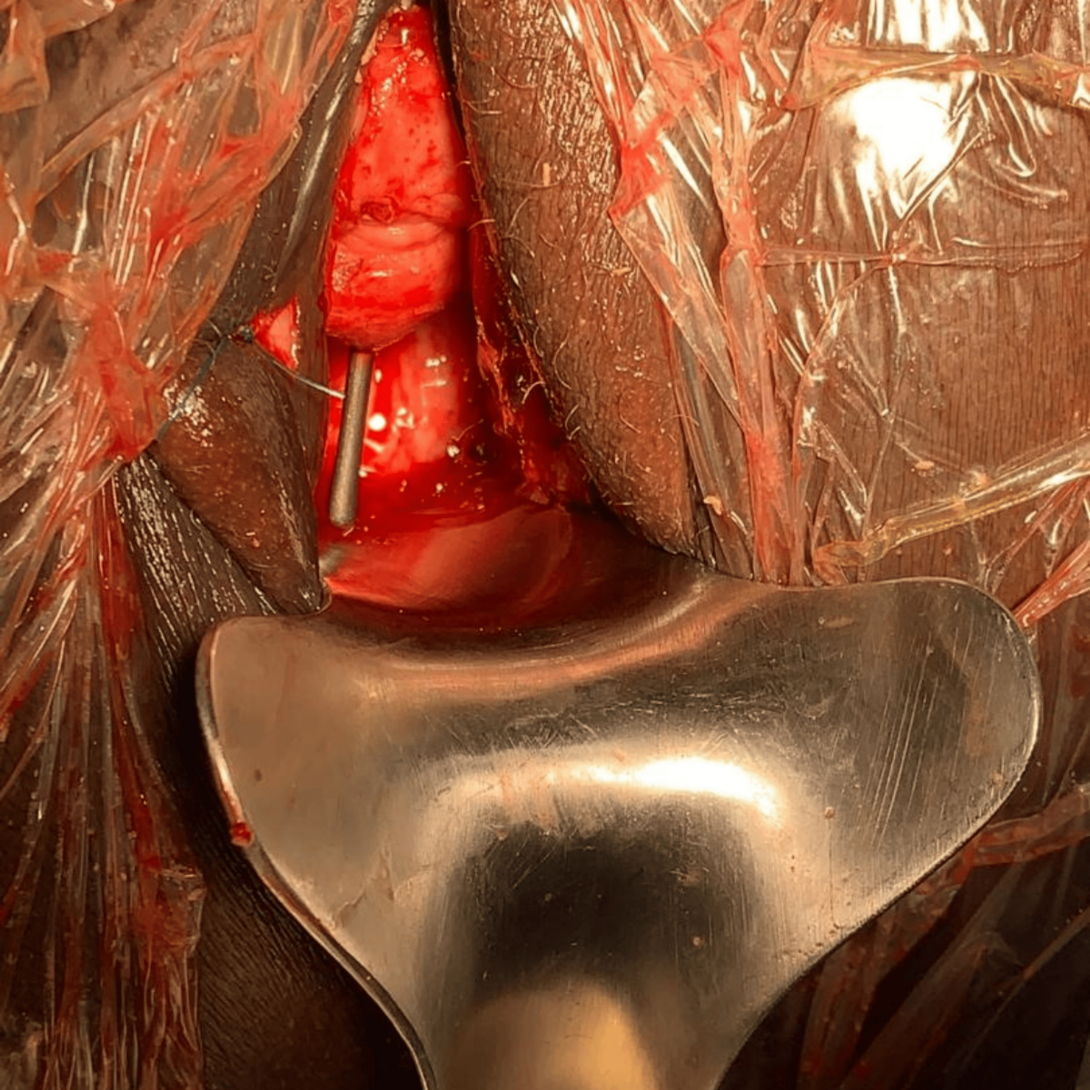
Vesicovaginal fistula identified after stone extraction.

Surgical intervention was indicated. A dual vaginal and suprapubic approach was performed. Due to the deep impaction of the stone, it was mechanically fragmented using bone forceps and then completely extracted vaginally and via cystolithotomy (Figure 2). After copious irrigation with saline solution and intraoperative assessment of tissue quality, the vesicovaginal fistula (Figure 3) was repaired during the same surgical procedure. The technique used was a split-suture approach, which consisted of separating the vagina from the bladder, excising the edges of the fistula, followed by separate closure of the bladder and then the vagina. No Martius flap was used. A urinary catheter was left in place postoperatively.

The postoperative course was uneventful. Bladder integrity was confirmed on day 30 and the patient regained complete urinary continence.

Written informed consent was obtained from the patient for the publication of this clinical case and associated images.

## Discussion

Vaginal foreign bodies can cause urogenital complications. The main clinical signs are vaginal discharge, metrorrhagia, pelvic pain, and often dyspareunia [4,5]. When their presence is unrecognized or concealed by patients, the symptoms can be misleading and delay diagnosis.

A vaginal tampon left in place for several years gradually calcified, forming a crystallization nucleus. The constant contact of this hardened mass with the anterior vaginal wall led to chronic tissue inflammation, tissue damage, and ischemia. Erosion of the vaginal and then bladder walls resulted in a vesicovaginal fistula. This phenomenon follows the same pathophysiological process as the development of pressure ulcers in cases of prolonged bed rest or obstetric fistulas due to dystocic delivery. We considered the possibility of a pre-existing fistula, but the patient did not complain of urinary incontinence in the months and years following childbirth; it was only 10 years postpartum that urinary leakage began.

Standard radiography, ultrasound, and computed tomography are imaging examinations necessary for diagnosis. Cystoscopy, vaginoscopy, or even MRI may be indicated in complex fistulas [6]. However, in some remote areas of sub-Saharan Africa, access to certain advanced imaging examinations during mobile obstetric fistula repair campaigns is limited. Diagnosis then relies primarily on clinical examination and, above all, intraoperative exploration. For large foreign bodies, as in our patient, imaging is not essential [7].

Fistula repair in two separate surgical stages, separated by at least three months, is recommended in cases of associated bladder stones [8,9]. In practice, in our setting, perfect microbiological control of the infection is not always possible: the priority is to ensure the absence of active signs of infection such as fever, sepsis, or pus. Good tissue vascularization is necessary. The absence of necrotic and friable tissue is also required. Finally, copious irrigation with isotonic saline and antibiotic prophylaxis or broad-spectrum empirical antibiotic therapy were performed.

Single-stage surgical repair is allowed when the infection is clinically controlled and tissue quality is deemed satisfactory. This reduces overall morbidity and the risk of loss to follow-up in areas with low hospital attendance rates. Other factors such as surgeon experience, surgical technique, fistula age, patient nutritional status, and comorbidities must also be considered for successful surgical repair [10,11]. Mengistu et al. reported in the literature a case of spontaneous closure of a vesicovaginal fistula after simple bladder catheterization maintained for two weeks [12]. These situations remain rare; the majority of fistulas require appropriate surgical treatment for lasting continence.

## Conclusions

Vesicovaginal fistula secondary to the presence of calcified foreign bodies in the vagina is rare. In the context of traveling fairs, a single-stage surgical procedure, based on rigorous intraoperative assessment, can offer excellent functional results.

Emphasis must be placed on educating patients in rural areas, raising awareness among healthcare workers about monitoring vaginal devices and safe obstetric practices to prevent these situations.
